# The Synthesis and Biological Evaluation of D-Ring-Modified Vitamin D Analogues

**DOI:** 10.3390/biom11111639

**Published:** 2021-11-04

**Authors:** Fumihiro Kawagoe, Sayuri Mototani, Atsushi Kittaka

**Affiliations:** Faculty of Pharmaceutical Sciences, Teikyo University, 2-11-1 Kaga, Itabashi, Tokyo 173-8605, Japan; fkawagoe@pharm.teikyo-u.ac.jp (F.K.); 19dy10003vu@stu.teikyo-u.ac.jp (S.M.)

**Keywords:** vitamin D_3_, synthesis, D-ring modification, structure-activity relationship

## Abstract

The vitamin D_3_ structure consists of the A-ring, a linker originating from the B-ring, C-ring, D-ring, and side-chain moieties. Each unit has its unique role in expressing the biological activities of vitamin D_3_. Many efforts have been made to date to assess the possible clinical use of vitamin D. Some organic chemists focused on the D-ring structure of vitamin D and synthesized D-ring-modified vitamin D analogues, and their biological activities were studied. This review summarizes the synthetic methodologies of D-ring-modified vitamin D analogues, except for *seco*-D, and their preliminary biological profiles.

## 1. Introduction

Vitamin D_3_ is a fat-soluble vitamin produced from 7-dehydrocholesterol through a two-step pericyclic reaction on the B-ring: a photochemical electrocyclic reaction and a subsequent thermal [1,7]-sigmatropic rearrangement. 25-Hydroxyvitamin D_3_ [25(OH)D_3_ (**1**)] and 1α,25-dihydroxyvitamin D_3_ [1α,25(OH)_2_D_3_ (**2**)] are important compounds responsible for various physiological functions to maintain homeostasis in the human body. Vitamin D_3_ is divided into five constructive units: An A-ring, a linker originating from the B-ring, a C-ring, a D-ring, and a side-chain, each of which plays an essential role in expressing vitamin D-related activities [1,2].

**Scheme 1:**
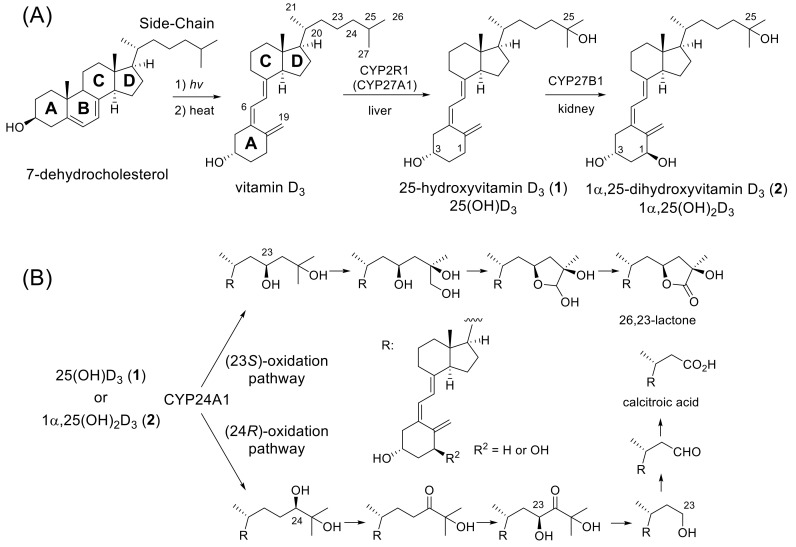
(**A**) 7-Dehydrocholesterol isomerism to vitamin D_3_ via a two-step sequential pericyclic reaction and its activation pathway to 25(OH)D_3_ (**1**) and 1α,25(OH)_2_D_3_ (**2**). (**B**) Deactivation pathways of **1** and **2** to 26,23-lactone and calcitroic acid through (23*S*)-oxidation and (24*R*)-oxidation, respectively.

Among these structures, both the A-ring and side-chain, in particular, are vitamin D_3_ activation and inactivation sites controlled by vitamin D-metabolizing CYPs [3,4,5], and the introduction of a substituent to and the structural transformation of these sites have been vigorously pursued. On the other hand, the CD-ring does not undergo direct hydroxylation in the activation and inactivation steps of the vitamin D_3_ molecule (Scheme 1). 

For the synthesis of vitamin D_3_ analogues, the CD-ring part is often used directly from the natural vitamin D_3_ CD-ring part due to the difficulty of constructing the chiral CD-ring structure. Therefore, it is challenging to introduce functional groups to this part or transform the CD-ring to another skeleton. As a result, the synthesis of vitamin D_3_ analogues with a modified CD-ring and evaluation of their biological activities are still less explored than synthesis of vitamin D analogues with a modified A-ring or side-chain. However, organic chemists have attempted the introduction of substituents and/or structural transformation of both C- and D-ring parts and have succeeded in synthesizing a group of analogues and evaluating their biological activities to date [6,7,8,9,10]. The D-ring unit is characterized by the direct attachment of the side chain, and there have been many reports that the introduction of substituents to or structural transformation of the D-ring has significant effects on vitamin D activities.

In this review, we focus on the D-ring, which is one of the essential sites forming the basic framework of the vitamin D_3_ molecule. We describe the synthetic approach to vitamin D_3_ analogues with substituents or structural transformation on the D-ring, except for *seco*-D analogues, and their preliminary biological activities. 

## 2. 16-Ene-Vitamin D_3_ Analogues

Among D-ring-modified vitamin D_3_ analogues, a wide variety of 16-ene-vitamin D_3_ analogues have been synthesized and evaluated regarding their biological properties as potential anticancer agents with low calcemic effects. 

**Scheme 2:**
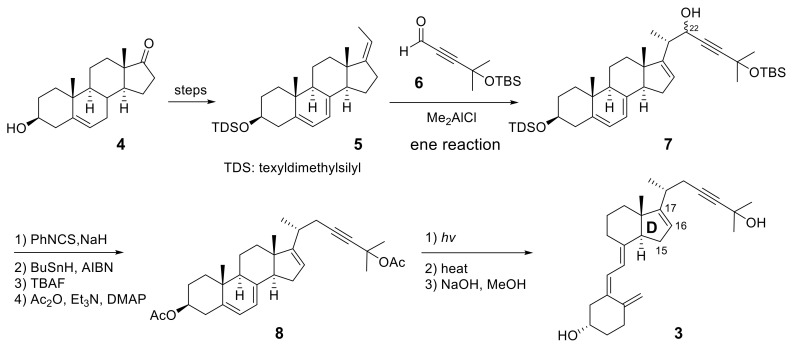
Synthesis of the first 16-ene-25-hydroxyvitamin D (**3**) via an ene reaction from sterol (**4**).

The first synthetic report on the 16-ene-vitamin D_3_ analogue (**3**) was described by Hoffmann-La Roche’s group in 1995 [11], and its biological activities were studied [12,13,14,15]. The synthetic route started from a commercially available steroid, dehydroepiandrosterone (**4**). The 16-ene unit was constructed utilizing an ene reaction between *Z*-olefin (**5**) and aldehyde (**6**) in the presence of Me_2_AlCl to give a mixture of C22-diastereomeric alcohols (**7**), and subsequent Barton deoxygenation afforded 16-en-23-yne (**8**). To construct the triene structure, photochemical conversion and subsequent thermal isomerization were applied on the B-ring diene system (Scheme 2). The final product, **3**, was identified as a potential antipsoriatic agent at that time.

**Scheme 3:**
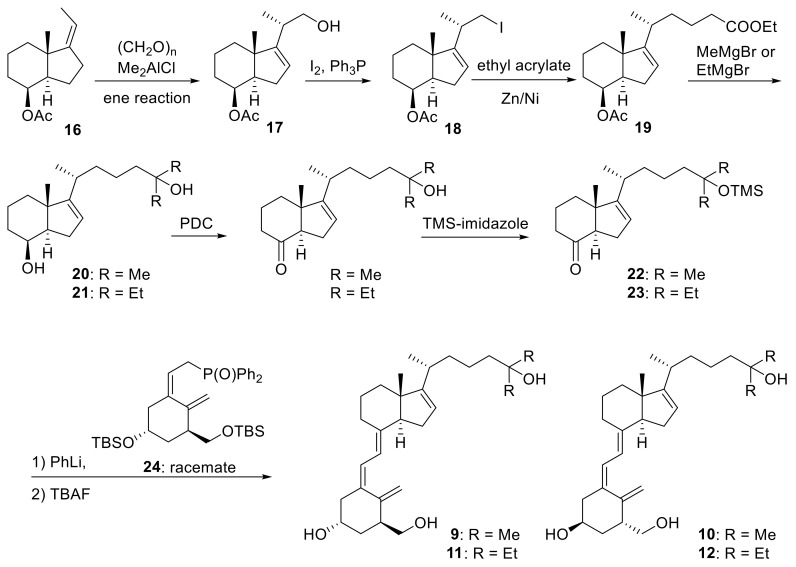
Posner’s approach to 1-hydroxymethyl-25-hydroxy-16-ene-vitamin D_3_ analogues (**9**–**12**) using the Wittig–Horner reaction.

**Scheme 4:**
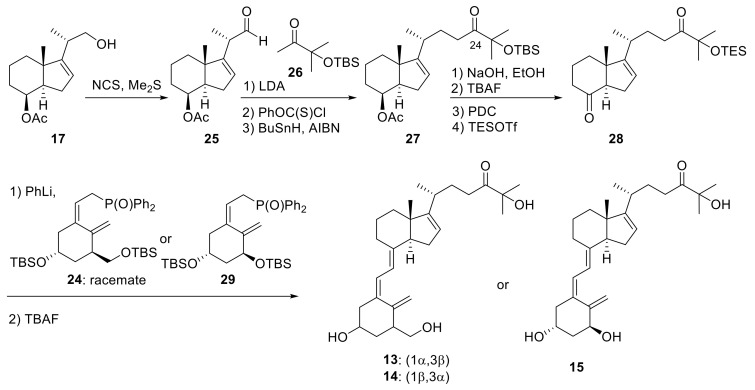
Synthesis of 24-oxo-16-ene-vitamin D_3_ analogues.

Posner and coworkers reported the synthesis of 16-ene-vitamin D_3_ analogues with the 1-hydroxymethyl group (**9–14**) and 24-oxo-16-ene vitamin D_3_ analogue (**15**) in 1997 (Scheme 3 and Scheme 4) [16,17].

In Scheme 3, the synthesis of 1-hydroxymethyl analogues (**9–12**) is described. Introduction of the key 16-ene unit was accomplished by a Me_2_AlCl-mediated ene reaction between olefin (**16**) and formaldehyde to afford alcohol (**17**). Next, **17** was converted to iodide (**18**), followed by the Zn/Ni-promoted Michael addition of iodide (**18**) to ethyl acrylate to produce ethyl ester (**19**). The ethyl ester (**19**) was treated with methyl magnesium bromide or ethyl magnesium bromide to give alcohol (**20**,**21**). After oxidation of **20** and **21**, protection of the C25-hydroxy group afforded 8-keto-CD-ring (**22**) and (**23**). Each ketone (**22**,**23**) and the racemic A-ring moiety (**24**) were coupled using PhLi, and then protecting groups of the coupling products were removed in the presence of TBAF to afford 16-ene analogues (**9–12**) (Scheme 3).

For the synthesis of 24-oxo-16-ene analogues (**13**–**15**), alcohol (**17**) was used as a starting material (Scheme 4). The synthesis began with the oxidation of primary alcohol to give aldehyde (**25**), followed by the addition of **26** to aldehyde (**25**) under basic conditions, to afford a mixture of C22-diastereomeric alcohols. Removal of the C22-hydroxy group using the Barton reaction yielded 24-oxo compound **27**, which was converted to 8-keto-CD-ring (**28**) in four steps. Coupling reactions using **28** and A-rings (**24**,**29**) were performed under the same conditions as in Scheme 3. Under the coupling conditions, the 24-oxo unit was inert, i.e., no chemical conversion was observed at the C24 position. 

**Figure 1:**
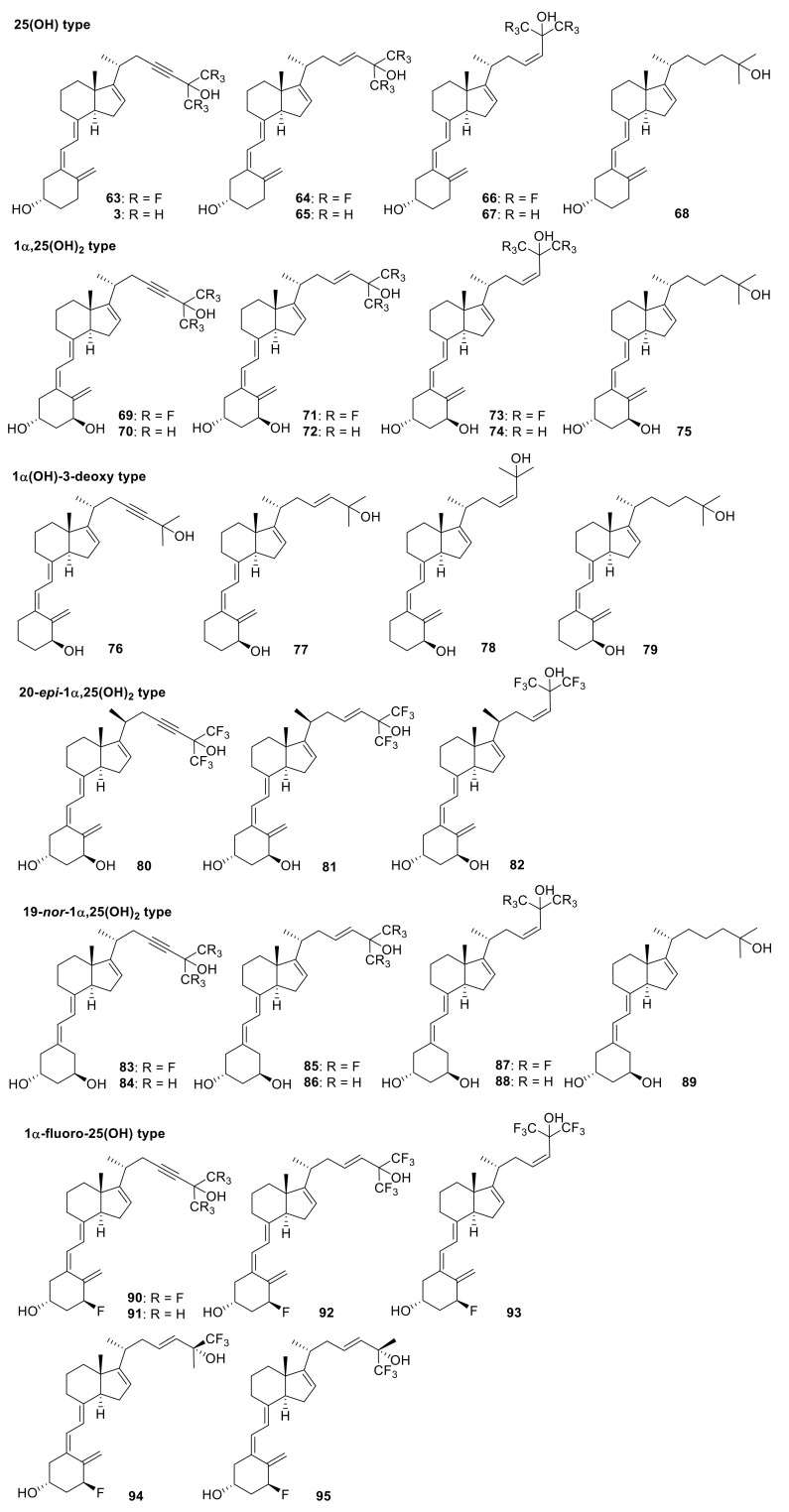
16-ene analogues from Uskoković’s group.

The authors evaluated the antiproliferative activity of the newly synthesized analogues (**10**,**12**,**14**,**15**) in murine keratinocytes in vitro and found that the 26,27-diethyl-1β-hydroxymethyl analogue **12** and the 24-oxo with a natural A-ring analogue **15** showed activity comparable with that of the natural hormone 1α,25(OH)_2_D_3_ (**2**), even at physiologically relevant nanomolar concentrations. The 22-oxo-analogue **15** was an intermediary metabolite of 16-ene-1α,25(OH)_2_D_3_ (**75** in Figure 1) formed through the (24*R*)-oxidation pathway (Scheme 1) with a longer half-life than the naturally occurring 24-oxo-1α,25(OH)_2_D_3_ and equipotent to parent **75** itself in regulating cell-growth and differentiation [18].

**Scheme 5:**
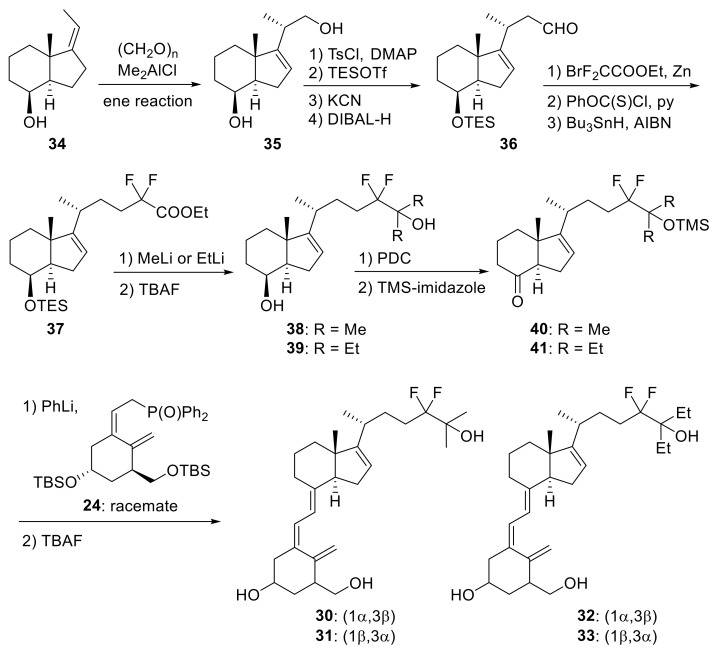
Synthesis of 24,24-difluoro-16-ene-1-hydroxymethyl-25-hydroxyvitamin D_3_ (**30**–**33**).

The same authors subsequently reported the 1-hydroxymethyl-24,24-difluoro-16-ene analogues (**30**–**33**) in 1998 [19]. The 16-ene-homoallylic alcohol (**35**) provided by an ene reaction of olefin (**34**) was first converted to tosylate via tosylation of the primary hydroxy group and triethylsilylation of the secondary hydroxy group. Tosylate was converted to nitrile upon treatment with KCN, followed by reduction with DIBAL-H to give aldehyde (**36**). Introduction of the 24,24-difluoro unit was accomplished with the Reformatsky reaction using ethyl bromodifluoroacetate and activated zinc powder. Treatment of ester (**37**) with methyl lithium or ethyl lithium, followed by fluoride-induced deprotection at the C-8 position generated the natural side-chain-CD-ring (**38**) and 26,27-dihomo-CD-ring (**39**), which were converted to 8-keto-CD-rings (**40**,**41**) in two steps. A Wittig–Horner coupling reaction between a racemic phosphine oxide (**24**) and the 8-keto-CD-rings (**40**,**41**), followed by deprotection of the silyl protecting groups, produced the target analogues (**30**–**33**) (Scheme 5).

The 1β-analogues (**31**,**33**) showed significant antiproliferative activity in murine keratinocytes and malignant melanoma cells, and this was equally potent to or even more potent than 1α,25(OH)_2_D_3_ (**2**). On the other hand, 1α-analogues (**30**,**32**) were much less potent than their 1β-counterparts (**31**,**33**). In addition, **31** and **33** showed no calcemic activity in vivo.

**Scheme 6:**
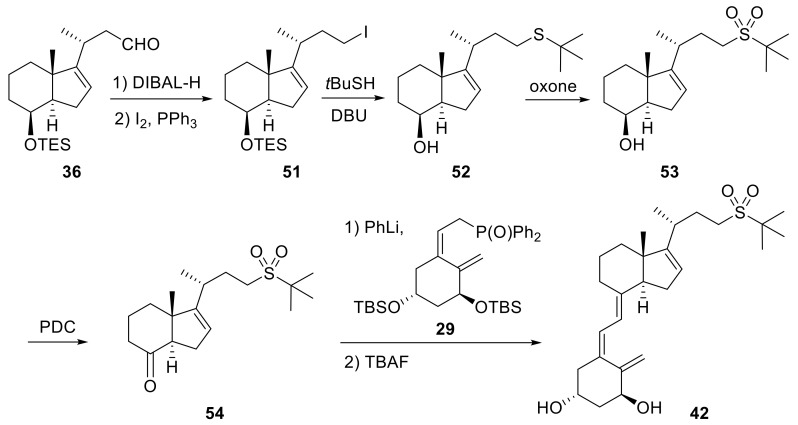
Synthesis of 23-*tert*-butylsulfonyl-16-ene-1α-hydroxyvitamin D analogue (**42**).

**Scheme 7:**
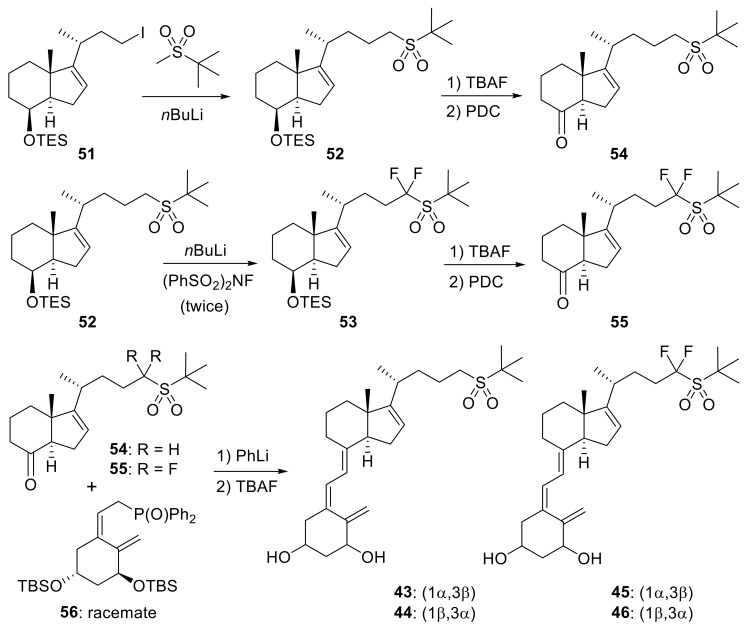
Synthesis of 24-*tert*-butylsulfonyl-16-ene-1-hydroxyvitamin D analogues (**43–46**).

**Scheme 8:**
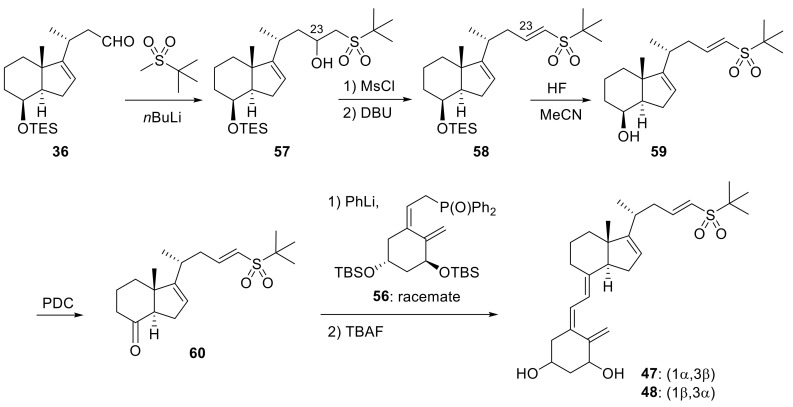
Synthesis of 24-*tert*-butylsulfonyl-16,23-diene-1-hydroxyvitamin D analogues (**47**,**48**).

**Scheme 9:**
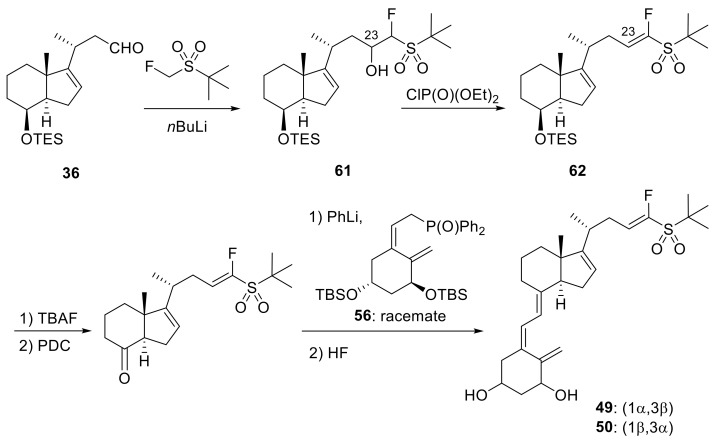
Synthesis of 24-*tert*-butylsulfonyl-16,23-diene-24-fluoro-1-hydroxyvitamin D analogues (**49**,**50**).

In 1999, 16-ene analogues with a sulfone unit (**42–44**,**47**,**48**) and their C24 fluorinated versions (**45**,**46**,**49**,**50**) were also reported (Scheme 6, Scheme 7, Scheme 8 and Scheme 9) [20].

For the synthesis of analogue **42**, the known aldehyde (**36**) was used as a starting material (Scheme 6). Reduction of aldehyde (**36**) and iodination of the primary alcohol yielded iodide (**51**). After conversion of the iodide to sulfide (**52**), oxidation of the sulfide using oxone provided sulfone (**53**), and subsequent PDC oxidation of the C8-OH group gave ketone (**54**). Finally, the convergent coupling reaction to form the desired analogue (**42**) was performed with the ketone (**54**) and A-ring (**29**) carbanion (Scheme 6).

Next, one-carbon-elongated analogues (**43**,**44**) and their C24-fluorinated versions (**45**,**46**) were prepared in a similar manner. Treatment of iodide (**51**) with *t*-butyl methyl sulfone under basic conditions gave a 25-sulfone CD-ring (**52**). Introduction of the difluoro unit to the C24 position of sulfone (**52**) was achieved by utilizing a cationic fluorination reagent in the presence of *n*BuLi to give **53**. Desilylation of **52** and **53**, followed by oxidation at the C8-OH group, afforded ketones (**54**,**55**). Finally, these ketones (**54**,**55**) were converted into the desired vitamin D_3_ analogues (**45–48**) using the convergent coupling reaction with a lithium anion of the racemic A-ring moiety (**56**) (Scheme 7).

Synthesis of 23-ene analogues (**47**,**48**) started from aldehyde (**36**) (Scheme 8). Treatment of **36** with *t*-butyl methyl sulfone under basic conditions afforded C23-OH adducts (**57**). Mesylation of C23-OH, followed by elimination gave olefin (**58**). Although deprotection of the TES protective group was problematic using TBAF because of the Michael addition of fluoride to the C23 position, HF-MeCN instead of TBAF gave **59** in a nearly quantitative yield. The resulting alcohol (**59**) was subjected to PDC oxidation to afford ketone (**60**). Finally, **60** was converted to the desired vitamin D_3_ analogues (**47**,**48**) using the same method as above in Scheme 7. 

Synthesis of 23-ene-24-fluoro analogues (**49**,**50**) is illustrated in Scheme 9. According to a similar methodology to that described in Scheme 8, an intermediate 23-hydroxy-24-fluoro-CD-ring (**61**) was prepared by the nucleophilic addition of *t*-butyl fluoromethyl sulfone to aldehyde (**36**) under basic conditions. Mesylation of the C23-hydroxy group and subsequent E2 elimination gave 23-ene-24-fluoro-olefin (**62**), which was converted to 23-ene-24-fluoro analogues (**49**,**50**) in four steps.

Antiproliferative activities of the newly synthesized 16-ene-vitamin D_3_ analogues (**42–45**,**47**,**49**) were tested in both murine keratinocytes and malignant melanoma cells in vitro. The data revealed that almost all analogues showed activities equivalent to or even stronger than those of 1α,25(OH)_2_D_3_ (**2**).

Next, the vitamin D receptor-mediated transcriptional activity of three analogues (**42**,**45**,**49**) in ROS 17/2.8 cells was evaluated, and **42** and **45** showed strong transcriptional activity only approximately twofold less active than that of 1α,25(OH)_2_D_3_ (**2**). For practical chemotherapeutic uses, the calcemic activity of five analogues (**42**,**43**,**45**,**47**,**49**) was tested in vivo, revealing that **42**, **45**, and **49** did not show calcemic activity at a 10 μg/kg dose for 1 week. In contrast, **47** was moderately calcemic, and **43** showed strong calcemic activity [20].

The 26,27-fluorinated-16-ene (**63**,**64**,**66**,**69**,**71**,**73**,**80–83**,**85**,**87**,**90**,**92**–**95**) and non-fluorinated (**3**,**65**,**67**,**68**,**70**,**72**,**74–79**,**84**,**86**,**88**,**89**,**91**) analogues have been synthesized to date (Figure 1). Uskoković et al. published a comprehensive review article in this field, including synthetic routes and biological activities [21].

**Scheme 10:**
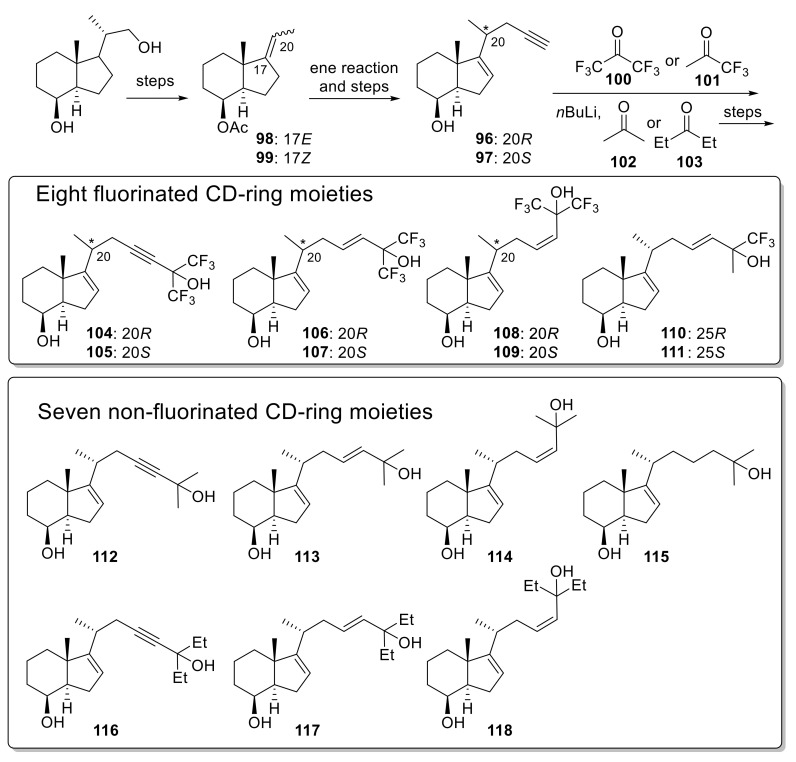
Synthesis of the 16-ene-CD-ring moieties (**104**–**118**). *: an asymmetric carbon atom.

Briefly, the preparation of 16-ene-CD-ring moieties (**104–118**) is illustrated in Scheme 10. First, key intermediates (**96**,**97**) were synthesized from olefins (**98**,**99**), and then these were treated with hexafluoroacetone (HFA) (**100**) or 1,1,1-trifluoroacetone (**101**) under basic conditions to afford HFA-adducts and 26,26,26-trifluoroacetone-adducts, respectively. Hydrogenation of the 23,24-triple bond of the HFA-adducts with Lindlar catalyst gave 23,24-*Z*-olefins (**108**,**109**). On the other hand, reduction of the HFA-adducts and 26,26,26-trifluoroacetone-adducts with LiAlH_4_ in the presence of NaOCH_3_ afforded 23,24-*E*-olefins (**106**,**107**,**110**,**111**). Seven non-fluorinated CD-rings (**112–118**) were prepared using the same methodology. These synthetic CD-ring precursors (**104–118**) were coupled with the A-ring moieties to give 16-ene analogues (**3**,**63–95**) in Figure 1.

These 16-ene analogues possessed potential activity to induce HL-60 cell differentiation. The 1α-hydroxy analogues **(69–75,80–89**) showed stronger activity (IC_50_, 0.1–1.6 nM) than 1-deoxy analogues (**3**,**63–68)** (25– >1000 nM), 3-deoxy analogues (**76–79**) (6.5–50.0 nM), and 1α-fluoro analogues **(90–95)** (1.8–12.0 nM) [21,22]. 

**Scheme 11:**
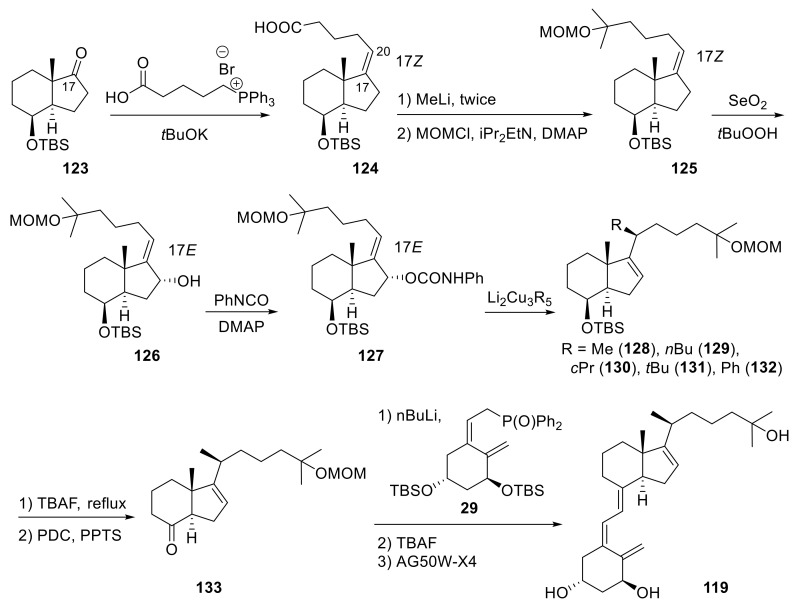
Mouriño’s convergent approach to C20-*epi*-16-ene-1α,25-dihydroxyvitamin D_3_ (**119**) using *syn*-S_N_2’ reaction as a key step.

**Scheme 12:**
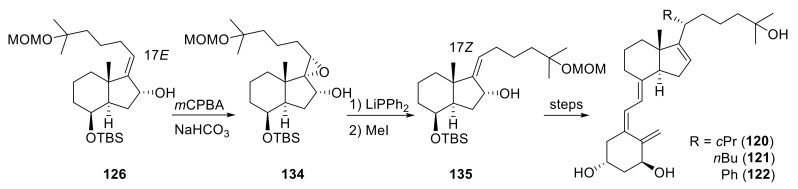
Construction of 17*Z*-CD-ring (**135**) using the Vedejs method and synthesis of 16-ene analogues (**120**–**122**).

Mouriño and coworkers described convergent synthetic routes for 16-ene-vitamin D_3_ analogues (**119–122**) in 1999 (Scheme 11 and Scheme 12) [23].

The synthesis of a C20-*epi* form of the 16-ene analogue (**119**) is shown in Scheme 11. The known C17-ketone (**123**) was used as a starting material, and stereoselective introduction of the 17*Z*-double bond was accomplished by a Wittig reaction. Treatment of carboxylic acid (**124**) with methyl lithium twice, followed by protection of the resulting C25-hydroxy group, afforded the desired CD-ring (**125**). The stereoselective oxidation of **125** with selenium dioxide afforded a C16-hydroxy-CD-ring (**126**). The C16-hydroxy group was subsequently converted to a leaving group, and the *syn*-S_N_2’ type reaction using a higher-order lithium cuprate (Li_2_Cu_3_R_5_, R = Me, *n*Bu, *c*Pr, *t*Bu, Ph) gave varieties of C20-*epi*-16-ene-CD-rings (**128–132**). Removal of the TBS protective group of **128** and subsequent PDC oxidation afforded ketone (**133**). Synthesis of a C20-*epi*-16-ene analogue (**119**) with the triene structure was achieved by the convergent method. 

It is possible to construct C20 natural form 16-ene-CD-rings using 17*Z*-carbamate (**135**) for a S_N_2’ *syn*-displacement reaction (Scheme 12). The key step for the C17-20 double-bond conversion from 17*E* (**126**) to 17*Z* (**135**) was accomplished by the Vedejs method [24]. Stereoselective epoxidation of **126**, followed by the addition of lithium diphenylphosphine and subsequent elimination, gave 17*Z*-CD-ring (**135**). From **135**, C20 natural type 16-ene analogues (**120–122**) were prepared, as shown in Scheme 11 (Scheme 12).

**Scheme 13:**
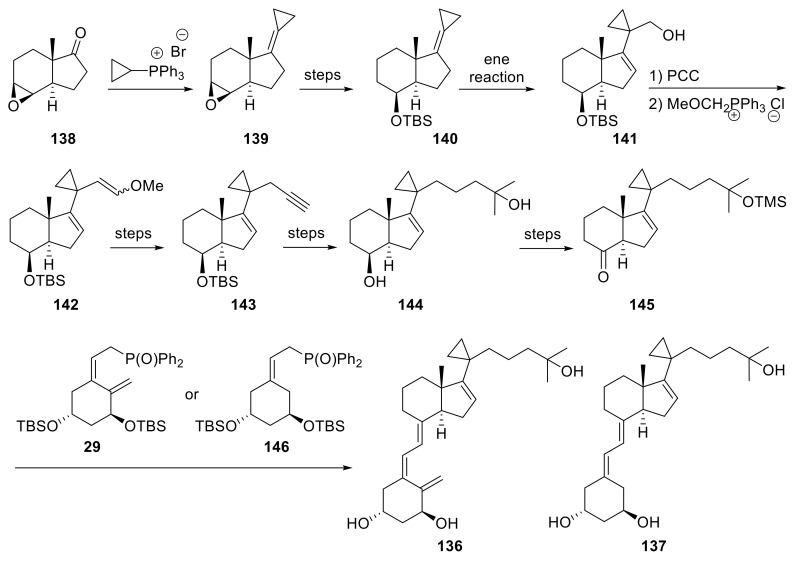
Synthesis of C20-cyclopropyl-16-ene analogues (**136**,**137**).

The synthesis and biological activities of C20-cyclopropyl-16-ene-1α,25-dihydroxyvitamin D_3_ analogues (**136**,**137**) were reported by Uskoković et al. in 2006 [25]. As shown in Scheme 13, **136** and a 19-nor type of **137** were prepared from ketone **138**. After introduction of the cyclopropyl unit by the Wittig reaction, reduction and TBS protection gave **140**. The 16-ene unit formation was accomplished by an ene reaction with formaldehyde to afford **141**. The alcohol **141** was subsequently oxidized with pyridinium chlorochromate, and the formed aldehyde was converted to alkyne **143** in three steps. Acetylide prepared from alkyne **143** was reacted with acetone, followed by deprotection of the *O*-TBS group and reduction of the alkynyl moiety to give C25-OH-CD-ring **144**. Oxidation of the C8-OH group, followed by trimethylsilylation of the C25-OH group, afforded 8-ketone **145**. Finally, synthesis of 16-ene analogues (**136**,**137**) was achieved in a convergent manner by connecting with A-ring (**29** or **146**). 

Immunomodulatory activity of C20-cyclopropyl-16-ene-1α,25-dihydroxyvitamin D_3_ analogues (**136**,**137**), the parent analogue (C20-cyclopropyl-1α,25(OH)_2_D_3_) (**147**), and 1α,25(OH)_2_D_3_ (**2**) was tested by suppression of interferon-γ (IFN-γ) release. Analogue (**136**) was found to be 45-times more potent than its parent analogue (**147**) and also 2440-times more active than 1α,25(OH)_2_D_3_ (**2**).

**Figure 2:**
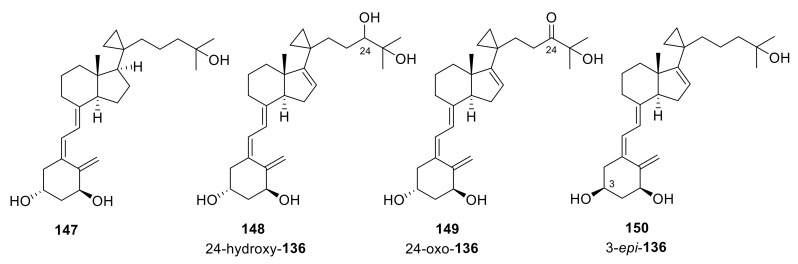
Structures of the 16-ene analogues (**147–150**) with the cyclopropyl unit at the C20 position.

A metabolism study of **136** using the rat osteosarcoma cell line (UMR106) revealed that three main metabolites were detected from the HPLC profiles. Comparing the metabolic studies of **136** and **147**, the authors concluded that these three metabolites were 24-hydroxy-**136** (**148**), 24-oxo-**136** (**149**), and 3-*epi*-**136** (**150**) (Figure 2).

**Scheme 14:**
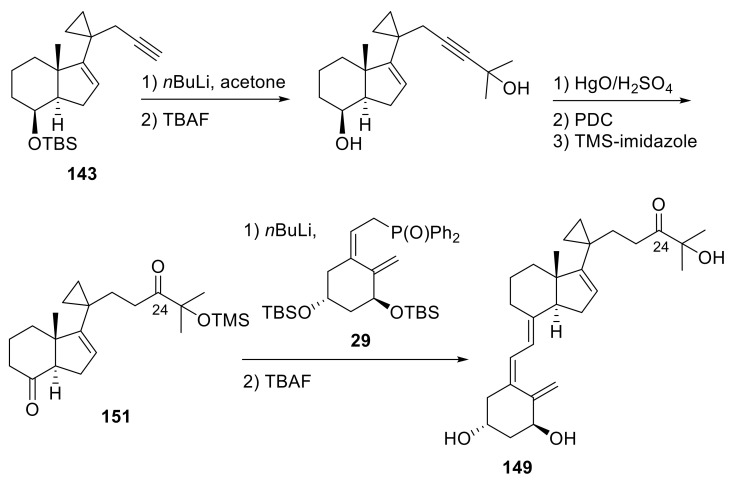
Synthesis of 16-ene-24-oxo analogue (**149**).

In 2009, the 16-ene-24-oxo-1α,25-dihydroxyvitamin D_3_ (**149**) was synthesized from the key intermediate **143** by a sequence of reactions illustrated in Scheme 14 [26]. Acetylide from alkyne **143** was reacted with acetone, followed by hydration of the resulting alkyne moiety using HgO in the presence of H_2_SO_4_ to obtain 24-oxo-CD-ring (**151**). The 24-oxo-CD-ring (**151**) was subsequently coupled with the A-ring (**29**) to give 16-ene-24-oxo-1α,25-dihydroxyvitamin D_3_ (**149**). 

The authors compared the VDR-dependent *CYP24A1* and cathelicidin antimicrobial peptide (*CAMP*) transcriptional activities of the 24-oxo analogue (**149**), its metabolic precursor **136**, and the natural hormone, 1α,25(OH)_2_D_3_ (**2**), using peripheral blood mononuclear cells (PBMCs) as well as human acute monocytic leukemia cells (THP-1 cells), and revealed that both 16-ene analogues (**136**,**149**) exhibited similar transcriptional activities (EC_50_ ~0.7 nM) and greater activities than the natural hormone, 1α,25(OH)_2_D_3_ (**2**) (EC_50_ ~10 nM). 

To compare the anti-inflammatory properties of **136**, **149**, and **2**, the authors tested their potency to inhibit the production of cytokines (IFN-γ, TNF-α, IL-12/23p40, and IL-6) in vitro, and concluded that **136** and its 24-oxo metabolite **149** had a similar activity and significantly higher potency compared to the natural hormone (**2**).

Evaluation of the calcemic activity of **136**, **149**, and **2** revealed that **136** and **149** were less calcemic than **2**, and **149** was the least calcemic among the three analogues.

## 3. 16-Modified Vitamin D_3_ Analogues

**Scheme 15:**
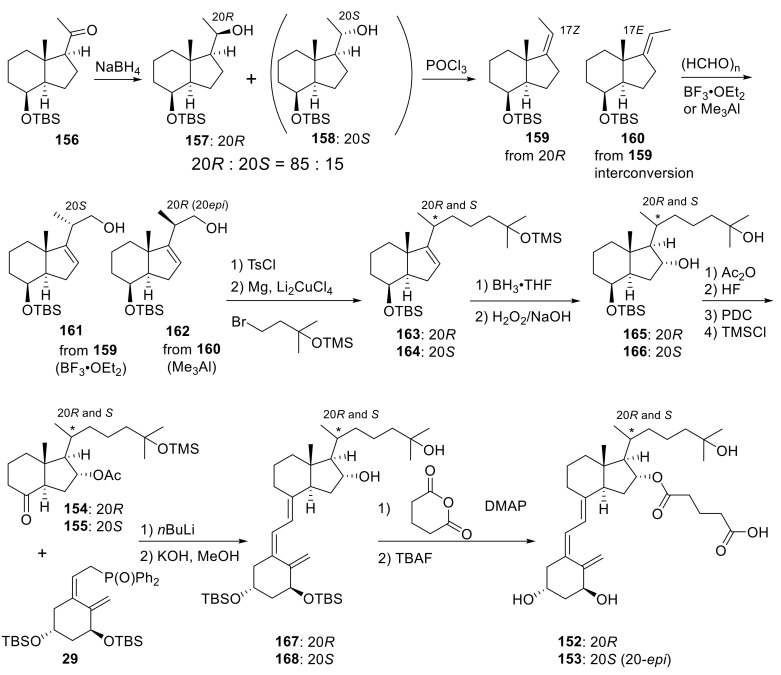
Synthesis of the C16-substituted analogue **152** and its C20-*epi*-version **153** for the hapten formula. *: an asymmetric carbon atom.

In 2003, the C16-substituted analogue **152** and its C20-*epi*-version **153** were reported by the LEO Pharma group in order to produce specific antibodies to 1α,25(OH)_2_D_3_ (**2**) and its C20-*epi* form (Scheme 15) [27]. The CD-ring synthons **154** and **155** were constructed from the known ketone **156**. Reduction of **156** with NaBH_4_ yielded a mixture of (20*R*)-OH-CD-ring (**157**) and its 20*S*-isomer (**158**) in a ratio of 85:15. After separation of each isomer, **157** and **158** were converted to C20-natural form CD-ring (**161**) and C20-*epi* form CD-ring (**162**), respectively, in two steps. Tosylation of **161** and **162**, followed by nucleophilic substitution to give **163** and C20-*epi* **164**. The key step for introducing the C16-OH group was achieved by hydroboration/oxidation of **163** and C20-*epi* **164** to afford C16α-OH **165** and C20-*epi*-16α-OH **166** along with C16β-OH isomers as the minor products. Both C16α-OH-CD-rings (**165**,**166**) were converted to 8-keto-CD-ring (**154**) and C20-*epi*-8-keto-CD-ring (**155**), respectively, in four steps. The Wittig–Horner coupling reaction with the lithium salt of the A-ring precursor **29** gave the coupling products. The acetate group of the coupling products was replaced with glutaric acid esters, followed by deprotection with TBAF to afford **152** and **153**.

Both analogues (**152**,**153**) were coupled with bovine serum albumin (BSA), and polyclonal antisera from the rabbits were obtained [28]. The antibodies to **152** had selective binding affinity for 1α,25(OH)_2_D_3_ (**2**) that was 12- to 13-times greater than for C20-*epi*-1α,25(OH)_2_D_3_. In contrast, antibodies to **153** exhibited poor selectivity between 1α,25(OH)_2_D_3_ (**2**) and C20-*epi*-1α,25(OH)_2_D_3_.

**Scheme 16:**
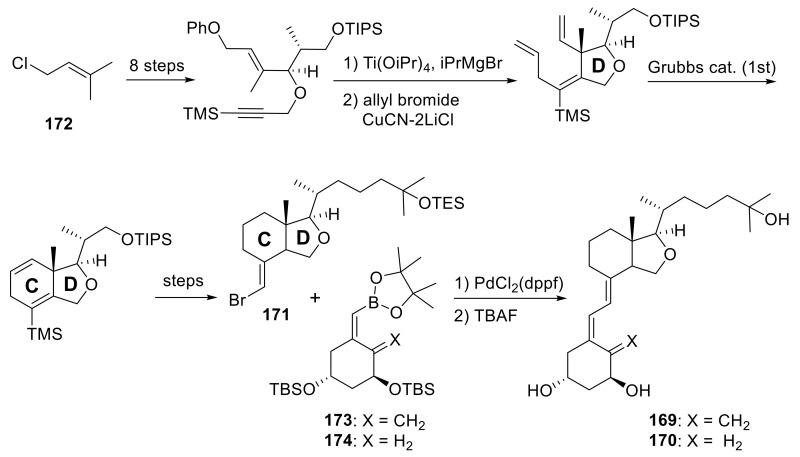
Synthesis of 16-oxa-1α,25-dihydroxyvitamin D_3_ and its 19-nor counterpart (**169**,**170**).

In 2019, Okamoto and coworkers reported the synthesis of 16-oxa-vitamin D_3_ analogues (**169**,**170**) [29]. They synthesized the 16-oxa-CD-ring moiety (**171**) starting from 1-chloro-3-methylbut-2-ene (**172**) in 20 steps (Scheme 16). For the construction of CD-ring parts, a Ti(II)-mediated enyne cyclization/Cu-catalyzed allylation and a subsequent ring-closing metathesis reaction (RCM) were applied. The synthetic **171** was coupled with the A-ring moieties (**173**,**174**) in the presence of PdCl_2_(dppf) and KOH to produce 16-oxa-vitamin D_3_ analogues (**169**,**170**). The VDR binding affinity of the newly synthesized 16-oxa analogues **169** and **170** was evaluated using the fluorescence polarization vitamin D-receptor competitor assay and time-resolved fluorescence resonance energy transfer VDR co-activator assay in comparison with those of the natural hormone (**2**) and its 19-nor form. It was demonstrated that both **169** and **170** were potent analogues. However, they were less potent than both the natural hormone (**2**) and its 19-nor-form.

## 4. Decalin-Vitamin D analogues

**Scheme 17:**
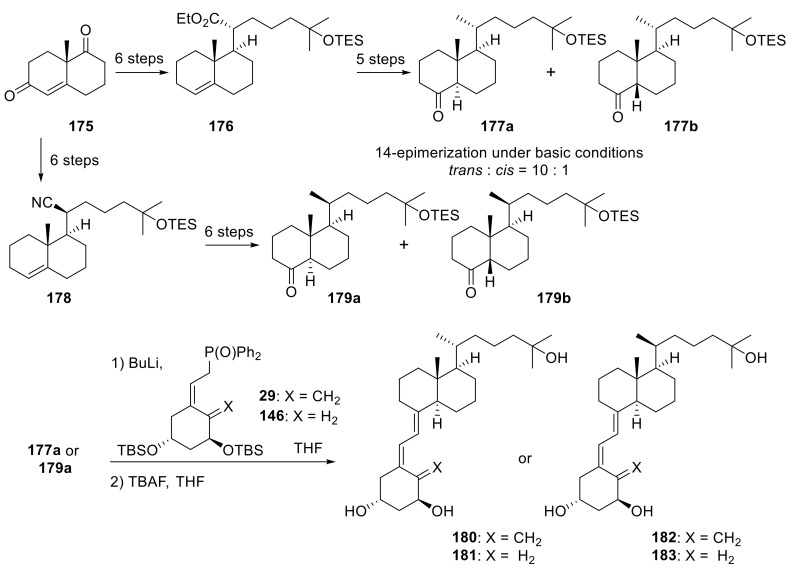
Synthesis of decalin-type 1α,25-dihydroxyvitamin D analogues (**180**,**182**) including 19-nor-analogues (**181**,**183**).

The natural hormone, 1α,25(OH)_2_D_3_ (**2**), contains a *trans*-hydrindane CD-ring fragment because of the biological synthetic pathway starting from lanosterol as the original steroidal skeleton. In 1996, Vandewalle et al. expanded the five-membered D-ring to a six-membered ring, and the CD-ring of the new vitamin D consisted of a *trans*-decalin ring system as shown in Scheme 17 [30]. (*S*)-Wieland–Miescher ketone **175** [31] was converted to *trans*-5-oxo-decalin **177a** with the natural 20*R*-configuration at the vitamin D_3_ side chain and also **179a** with the unnatural 20*S*-configuration side chain. During the synthesis of **177a** and **179a**, *cis*-fused decalins (**177b**, **179b**) were also produced; however, treatment of the mixture of 5-oxo-decalins under basic conditions afforded *trans*-decalins as the major products in their equilibriums, respectively. These decalin-type CD-rings were connected to the A-ring precursors (**29** or **146**), respectively, to yield the new class of 1α,25(OH)_2_D_3_ analogues **180** and **182** as well as the 19-nor-versions **181** and **183**. It is noteworthy that the natural 20*R*-configuration analogues of **180** and **181** showed higher VDR binding affinity, which was almost at the same level as that of 1α,25(OH)_2_D_3_ (**2**), than their 20-*epi*-counterparts **182** and **183**, and the cell differentiation and proliferation activity of **180** and **181** was one order of magnitude higher than that of **2**; however, 20-*epi*-counterparts **182** and **183** possessed weaker activity than **2**. The *trans*-decalin pair represented one of the rare examples that the 20-epimer exhibited reduced biological activities when compared with the analogues with the natural 20*R*-configuration [32].

**Scheme 18:**
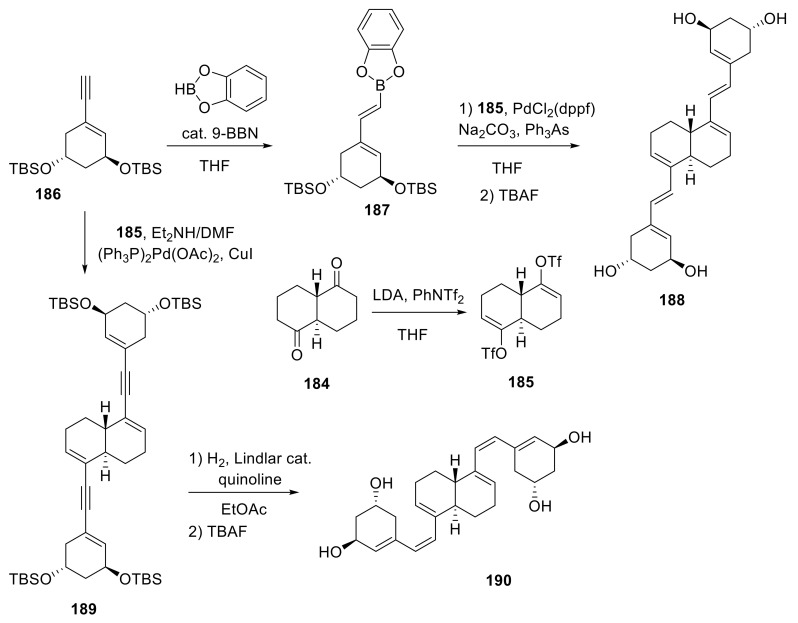
Synthesis of pseudo-*S*_2_-symmetrical vitamin D analogues with decalin core (**188**,**190**).

De Clercq’s group developed the *trans*-decalin core structure for *pseudo*-*S*_2_-symmetrical vitamin D analogues (**188**,**190**) [33]. For the synthesis, bis-triflate **185** was prepared from crystalline diketone **184** [34], and enyne **186** was connected in two-ways using Suzuki–Miyaura coupling and Sonogashira coupling reactions followed by appropriate chemical treatments to afford **188** and **190** in crystalline form, respectively (Scheme 18). However, these analogues did not show VDR-binding and antiproliferative activities.

**Figure 3:**
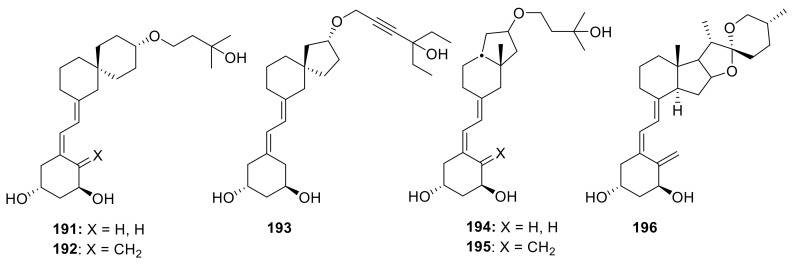
Unique examples of the modified D-ring moiety (**191–196**).

As shown in Figure 3, the De Clercq group also developed various CD-ring modified analogues **191–195 [35,36,37]**. These spiro-analogues showed low calcemic activity. From a natural product diosgenin, a spiro-vitamin D analogue **196** was synthesized by a Japanese group in 2005, and this compound, **196**, induced apoptosis in Hep G2 cells through activation of *p53* and *Bax* mRNAs [38]. 

Verlinden et al. published excellent review articles on CD-ring modified analogues of vitamin D, which included a D-ring- or C-ring lacking vitamin D molecules, and interestingly, these analogues showed moderate vitamin-D-related biological activities [39,40].

## 5. 15-Substituted Vitamin D_3_ Analogues

15-Hydroxyvitamin D_3_ and its derivatives were synthesized and reported by our group in 2011; i.e., 1α,15α,25-trihydroxyvitamin D_3_ (**212**) and 15α-methoxy-1α,25(OH)_2_D_3_ (**213**) as well as the 16-ene versions (**220**,**221**) were designed, synthesized, and biologically tested [41].

**Scheme 19:**
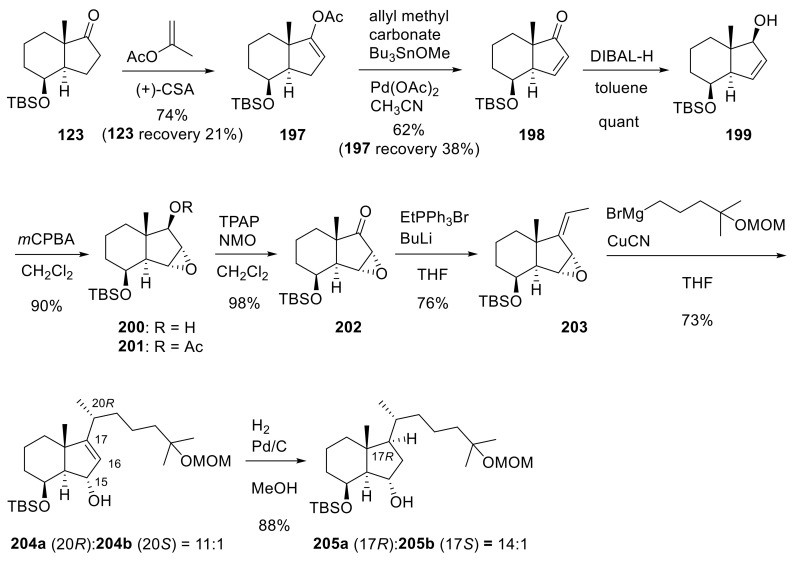
Synthesis of 15-substituted CD-rings (**204**,**205**).

As shown in Scheme 19, vinyl acetate **197** from the known ketone **123** through transacetylation using isopropenyl acetate reacted with allyl methyl carbonate in the presence of Pd(OAc)_2_ and Bu_3_SnOMe to afford α,β-unsaturated ketone **198**. The enone **198** was reduced with DIBAL-H to give allyl alcohol **199,** stereoselectively. Next, *m*CPBA epoxidation afforded α-epoxide **200**, whose stereochemistry was determined by X-ray crystallographic analysis of its acetate **201**. Oxidation of **200** afforded 17-oxo-α-epoxide **202**, and a Wittig reaction gave (17*Z*)-ethylidene **203** as a single isomer, although it was reported that 17-oxo-β-epoxysterols afforded only (17*E*)-ethylidene by the corresponding Wittig reaction [42,43,44]. 5-Bromo-2-methyl-2-pentanol MOM ether was converted to magnesium cyanocuprate, and 1,4-addition to ethylidene epoxide **203** yielded the 15α-hydroxy-16-ene-CD ring **204** with natural 20*R-* and unnatural 20*S*-configurations in a ratio of 11:1. The major isomer **204a** results from attack of the alkyl cuprate on the β-face of (17*Z*)-alkene **181** in an S_N_2’ manner. The D-ring double bond of **204a** was hydrogenated to afford **205** with a natural 17*R-* and unnatural 17*S*-configuration in a ratio of 14:1.

**Scheme 20:**
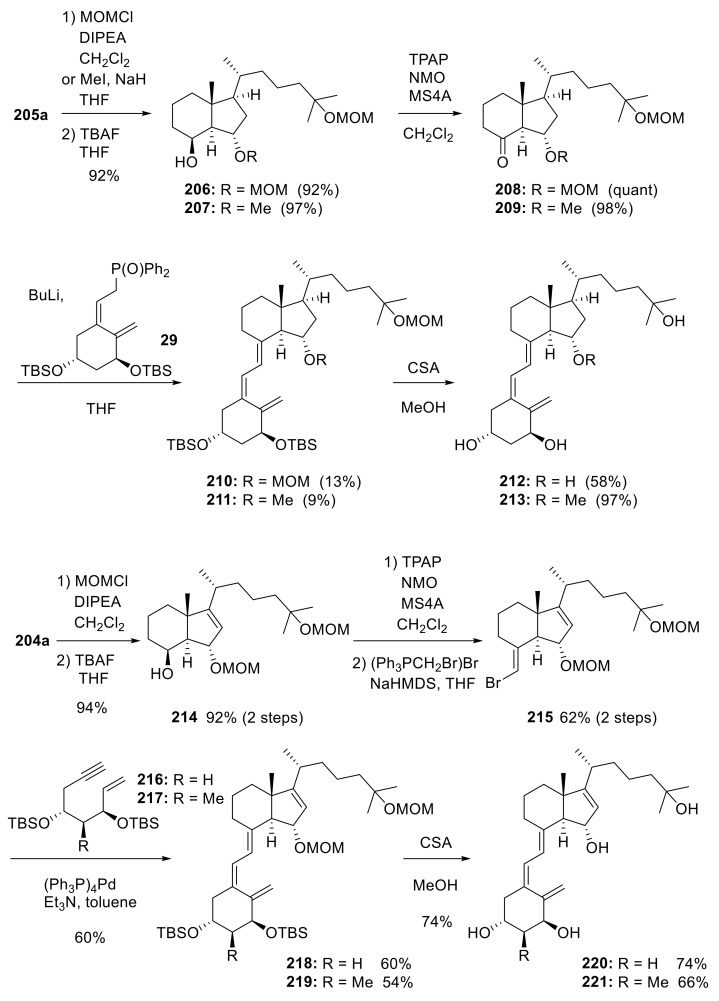
Synthesis of 15-substituted-1α,25-dihydroxyvitamin D_3_ and its 16-ene analogues (**212**,**213**,**220**,**221**).

The secondary hydroxy group at C15 of **205a** was methoxymethylated (**206**) or methylated (**207**), and C8-OH oxidation after deprotection afforded C8-ketone (Scheme 20). Ketones **208** and **209** were connected with A-ring phosphine oxide **29** by the Wittig–Horner reaction to yield the coupling products **210** and **211**, respectively. The subsequent deprotection gave the C15-modified analogues **212** and **213**. The C15-substituted 16-ene-vitamin D_3_ analogues were also available from **204a**, which was converted to bromoolefin, **215**, in four steps. A Trost coupling reaction with enyne **216** yielded 16-ene analog **218**, which was deprotected to afford the desired 15-hydroxy-16-ene-analogue **220**. To study the synergetic effects of the 2α-methyl group and the 16-ene-structure on biological activity, compound **221** was next synthesized using our original enyne **217** (Scheme 18) [45,46,47,48,49].

The basic biological activity of the novel compounds **212**, **213**, **220**, and **221** was tested: (1) VDR binding affinity was 13% (**212**), 1.5% (**213**), 65% (**220**), and 278% (**221**) of that of 1α,25(OH)_2_D_3_ (**2**). (2) EC_50_ values of transactivation activity of the osteocalcin promoter in HOS cells were 0.12, 0.12, 0.08, and 0.12 nM, respectively, with 0.09 nM of **2**. Interestingly, 16-ene analogs **220** and **221** exhibited comparable and even greater affinity for VDR and transactivation activity than the natural hormone (**2**) [41,50].

## 6. Conclusions

Actually, the 16-ene structure of the CD-ring part of vitamin D_3_ first appeared in the total synthesis of 1α,25(OH)_2_D_3_ (**2**) reported by the Hoffmann-La Roche group in 1982 [51]. To elongate the side chain at C20 from the (17*Z*)-ethylidene CD-ring creating the 20*R*-natural stereochemistry, they utilized the ene reaction with ethyl propionate in the presence of Lewis acid EtAlCl_2_. After the successful ene reaction, the first 16-ene structure was obtained, but the double bond was then reduced stereo-selectively to the saturated CD-ring system that was included in the natural active vitamin D_3_ skeleton. A great variety of unique 16-ene vitamin D analogs were synthesized using the ene reaction, and their biological activities were evaluated by Hoffmann-La Roche group and Posner’s group as shown in Section 2. It was proved that 16-ene-1α,25(OH)_2_D_3_ had higher binding affinity for the VDR, lower affinity for the vitamin D binding protein in circulation, and greater resistance to 24-oxo-mediated catabolism as compared with 1α,25(OH)_2_D_3_. Another strategy for 16-ene construction was developed by Mouriño’s group in 1999. They utilized S_N_2’-*syn*-facial displacement toward (17*Z*)-olefin carbamate from **135** by high-order cuprates (Li_2_Cu_3_Me_5_, etc.) to generate the 20*R*-natural configuration and the 16-ene double bond with high yield. The leaving group, a carbamate group, for the S_N_2’ reaction was located at the C16 position, and various types of substituents could be introduced at the C20 position, stereo-selectively, by this route. On the other hand, examples of 16-modified vitamin D analogues were few as described in Section 3, and only hydroxylated analogues were synthesized in which Mouriño’s synthetic intermediate was included as above. The unique example was Okamoto’s 16-oxa analogues of 1α,25(OH)_2_D_3_, even though these analogues exhibited weaker VDR binding affinity. Vandewalle and De Clercq developed a *trans*-decalin CD-ring system in the vitamin D structure, and these analogues showed potent biological activities, which is summarized in Section 4. Finally, our synthetic 15-substituted vitamin D_3_ analogues that possessed moderate biological activity were described in Section 5, but combination with 16-ene modification brought higher potency in VDR binding and osteocalcin transactivation activity than the natural hormone **2**. Although it is known that seco-D-ring system as vitamin D analogues [39], this review covers real ring systems as the vitamin D derivatives with the chemically modified D-ring. Modification on the vitamin D skeleton is worthy of challenging, and only real synthetic molecules are able to tell us benefits for the specific disease treatment.

## References

[1] Posner G.H., Kahraman M., Feldman D., Pike J.W., Glorieux F.H. (2005). Overview: Rational design of 1α,25-dihydroxyvitamin D_3_ analogs (Deltanoids). Vitamin D.

[2] Kulesza U., Sigüeiro R., Mouriño A., Sicinski R.R. (2013). Synthesis of 9-alkylated calcitriol and two 1α,25-dihydroxy-9-methylene-10,19-dihydrovitamin D_3_ analogues with a non-natural triene system by thermal sigmatropic rearrangements. J. Org. Chem..

[3] Rochel N., Wurtz J.M., Mitschler A., Klaholz B., Moras D. (2000). The crystal structure of the nuclear receptor for vitamin D bound to its natural ligand. Mol. Cell.

[4] Sakaki T., Sawada N., Komai K., Shiozawa S., Yamada S., Yamamoto K., Ohyama Y., Inouye K. (2000). Dual metabolic pathway of 25-hydroxyvitamin D_3_ catalyzed by human CYP24. Eur. J. Biochem..

[5] Sakaki T., Kagawa N., Yamamoto K., Inouye K. (2005). Metabolism of vitamin D_3_ by cytochromes P450. Front. Biosci..

[6] Yasuda K., Nishikawa M., Okamoto K., Horibe K., Mano H., Yamaguchi M., Okon R., Nakagawa K., Tsugawa N., Okano T. (2021). Elucidation of metabolic pathways of 25-hydroxyvitamin D_3_ mediated by CYP24A1 and CYP3A using *Cyp24a1* knockout rats generated by CRISPR/Cas9 system. J. Biol. Chem..

[7] Ordentlich P., Heyman R.A., Feldman D., Pike J.W., Glorieux F.H. (2005). Nonsteroidal Analogs. Vitamin D.

[8] Kulesza U., Plum L.A., DeLuca H.F., Mouriño A., Sicinski R.R. (2015). Novel 9-alkyl- and 9-alkylidene-substituted 1α,25-dihydroxyvitamin D_3_ analogues: Synthesis and biological examinations. J. Med. Chem..

[9] Gogoi P., Seoane S., Sigüeiro R., Guiberteau T., Maestro M.A., Pérez-Fernández R., Rochel N., Mouriño A. (2018). Aromatic-based design of highly active and noncalcemic vitamin D receptor agonists. J. Med. Chem..

[10] Zhou X., Zhu G.-D., Van Haver D., Vandewalle M., De Clercq P.J., Verstuyf A., Bouillon R. (1999). Synthesis, biological activity, and conformational analysis of four *seco*-D-15,19-*bisnor*-1α,25-dihydroxyvitamin D analogues, diastereomeric at C17 and C20. J. Med. Chem..

[11] Okabe M., Sun R.-C., Scalone M., Jibilian C.H., Hutchings S.D. (1995). Synthesis of 25-hydroxycholecalcifer-16-en-23-ynol: A potential antipsoriatic agent. J. Org. Chem..

[12] Zhou J.-Y., Norman A.W., Chen D.-L., Sun G.-W., Uskokovic M., Koeffler H.P. (1990). 1,25-Dihydroxy-16-ene-23-yne-vitamin D_3_ prolongs survival time of leukemic mice. Proc. Natl. Acad. Sci. USA.

[13] Jung S.A., Lee Y.Y., Pakkala S., de Vos S., Elstner E., Norman A.W., Green J., Uskokovic M., Koeffler H.P. (1994). 1,25(OH)_2_-16-ene-vitamin D_3_ is a potent antileukemic agent with low potential to cause hypercalcemia. Leuk. Res..

[14] Rao L.G., Sutherland M.K., Reddy G.S., Siu-Caldera M.-L., Uskokovic M.R., Murray T.M. (1996). Effects of 1α,25-dihydroxy-16ene, 23yne-vitamin D_3_ on osteoblastic function in human osteosarcoma SaOS-2 cells: Differentiation-stage dependence and modulation by 17-β estradiol. Bone.

[15] Chen T.C., Persons K., Uskokovic M.R., Horst R.L., Holick M.F. (1993). An evaluation of 1,25-dihydroxyvitamin D_3_ analogues on the proliferation and differentiation of cultured human keratinocytes, calcium metabolism and the differentiation of human HL-60 cells. J. Nutr. Biochem..

[16] Posner G.H., Lee J.K., White M.C., Hutchings R.H., Dai H., Kachinski J.L., Dolan P., Kensler T.W. (1997). Antiproliferative hybrid analogs of the hormone 1α,25-dihydroxyvitamin D_3_: Design, synthesis, and preliminary biological evaluation. J. Org. Chem..

[17] Honzawa S., Yamamoto Y., Yamashita A., Sugiura T., Kurihara M., Arai M.A., Kato S., Kittaka A. (2008). The 2α-(3-hydroxypropyl) group as an active motif in vitamin D_3_ analogues as agonists of the mutant vitamin D receptor (Arg274Leu). Bioorg. Med. Chem..

[18] Siu-Caldera M.-L., Clark J.W., Santos-Moore A., Peleg S., Liu Y.Y., Uskoković M.R., Sharma S., Reddy G.S. (1996). 1α,25-dihydroxy-24-oxo-16-ene vitamin D_3_, a metabolite of a synthetic vitamin D_3_ analog, 1α,25-dihydroxy-16-ene vitamin D_3_, is equipotent to its parent in modulating growth and differentiation of human leukemic cells. J. Steroid Biochem. Mol. Biol..

[19] Posner G.H., Lee J.K., Wang Q., Peleg S., Burke M., Brem H., Dolan P., Kensler T.W. (1998). Noncalcemic, antiproliferative, transcriptionally active, 24-fluorinated hybrid analogues of the hormone 1α,25-dihydroxyvitamin D_3_. Synthesis and preliminary biological evaluation. J. Med. Chem..

[20] Posner G.H., Wang Q., Han G., Lee J.K., Crawford K., Zand S., Brem H., Peleg S., Dolan P., Kensler T.W. (1999). Conceptually new sulfone analogues of the hormone 1α,25-dihydroxyvitamin D_3_: Synthesis and preliminary biological evaluation. J. Med. Chem..

[21] Uskoković M.R., Studzinski G.P., Gardner J.P., Reddy S.G., Campbell M.J., Koeffler H.P. (1997). The 16-ene vitamin D analogs. Curr. Pharm. Des..

[22] Uskoković M.R., Studzinski G.P., Reddy G.S., Feldman D., Glorieux F.H., Pike J.W. (1997). The 16-ene vitamin D analogs. Vitamin D.

[23] Rey M.A., Martínez-Pérez J.A., Fernández-Gacio A., Halkes K., Fall Y., Granja J., Mouriño A. (1999). New synthetic strategies to vitamin D analogues modified at the side chain and D ring. Synthesis of 1α,25-dihydroxy-16-ene-vitamin D_3_ and C-20 analogues. J. Org. Chem..

[24] Fuchs P.L., Vedejs E. (1971). Olefin inversion by the phosphorus betaine method. J. Am. Chem. Soc..

[25] Uskoković M.R., Manchand P., Marczak S., Maehr H., Jankowski P., Adorini L., Reddy G.S. (2006). C-20 Cyclopropyl vitamin D_3_ analogs. Curr. Top. Med. Chem..

[26] Laverny G., Penna G., Uskoković M., Marczak S., Maehr H., Jankowski P., Ceailles C., Vouros P., Smith B., Robinson M. (2009). Synthesis and anti-inflammatory properties of 1α,25-dihydroxy-16-ene-20-cyclopropyl-24-*oxo*-vitamin D_3_, a hypocalcemic, stable metabolite of 1α,25-dihydroxy-16-ene-20-cyclopropyl-vitamin D_3_. J. Med. Chem..

[27] Blæhr L.K.A., Björkling F., Calverley M.J., Binderup E., Begtrup M. (2003). Synthesis of novel hapten derivatives of 1α,25-dihydroxyvitamin D_3_ and its 20-epi analogue. J. Org. Chem..

[28] Hosoda H., Sakai Y., Yoshida H., Miyairi S., Ishii K., Nambara T. (1979). The preparation of steroid N-hydroxysuccinimide esters and their reactivities with bovine serum albumin. Chem. Pharm. Bull..

[29] Ibe K., Yamada T., Okamoto S. (2019). Synthesis and vitamin D receptor affinity of 16-oxa vitamin D_3_ analogues. Org. Biomol. Chem..

[30] Chen Y.-J., De Clercq P., Vandewalle M. (1996). Synthesis of new vitamin D_3_ analogues with a decalin-type CD-ring. Tetrahedron Lett..

[31] Lutz F.T., Utecht J. (1993). Improved preparation of enantiomerically pure (+)-(4a*S*,5*S*)-5-*tert*-butoxy-4a-methyl-4,4a,5,6,7,8-hexahydro-2(3*H*)-naphthalenone. Synthesis.

[32] Chen Y.-J., Gao L.-J., Murad I., Verstuyf A., Verlinden L., Verboven C., Bouillon R., Viterbo D., Milanesio M., Van Haver D. (2003). Synthesis, biological activity, and conformational analysis of CD-ring modified *trans*-decalin 1α,25-dihydroxyvitamin D analogs. Org. Biomol. Chem..

[33] Minne G., Verlinden L., Verstuyf A., De Clercq P.J. (2009). Synthesis of 1α,25-dihydroxyvitamin D analogues featuring a *S*_2_-symmetric CD-ring core. Molecules.

[34] Minne G.B., De Clercq P.J. (2007). Synthesis of 3,4,7,8-tetrahydronaphthalene-1,5(2*H*,6*H*)-dione. Molecules.

[35] Schepens W., Van Haver D., Vandewalle M., De Clercq P.J., Bouillon R., Verstuyf A. (2004). Synthesis and biological activity of 22-oxa CD-ring modified analogues of 1α,25-dihydroxyvitamin D_3_: Spiro[5.5]undecane CF-ring analogues. Bioorg. Med. Chem. Lett..

[36] De Buysser F., Verlinden L., Verstuyf A., De Clercq P.J. (2009). Synthesis of 22-oxaspiro[4.5]decane CD-ring modified analogs of 1α,25-dihydroxyvitamin D_3_. Tetrahedron Lett..

[37] Demin S., Van Haver D., Vandewalle M., De Clercq P.J., Bouillon R., Verstuyf A. (2004). Synthesis and biological activity of 22-oxa CD-ring modified analogues of 1α,25-dihydroxyvitamin D_3_: Cis-perhydrindane CE-ring analogues. Bioorg. Med. Chem. Lett..

[38] Quan H.-J., Koyanagi J., Komada F., Saito S. (2005). Preparations of vitamin D analogs, spirostanols and furostanols from diosgenin and their cytotoxic activities. Eur. J. Med. Chem..

[39] Verlinden L., Bouillon R., De Clercq P., Verstuyf A., Feldman D., Pike J.W., Bouillon R., Giovannucci E., Goltzman D., Hewison M. (2018). Analogs of Calcitriol. Vitamin D.

[40] De Clercq P.J., De Buysser F., Minne G., Schepens W., Vrielynck F., van Haver D., Vandewalle M., Verstuyf A., Bouillon R. (2007). The development of CD-ring modified analogs of 1α,25-dihydroxyvitamin D. J. Steroid Biochem. Mol. Biol..

[41] Shindo K., Kumagai G., Takano M., Sawada D., Saito N., Saito H., Kakuda S., Takagi K., Ochiai E., Horie K. (2011). New C15-Substituted Active Vitamin D_3_. Org. Lett..

[42] Takahashi T., Ootake A., Tsuji J., Tachibana K. (1985). Regio- and stereoselective introduction of 15β-hydroxy group and side chains to steroids by the palladium-catalyzed reaction of 1,3-diene monoepoxide. Tetrahedron.

[43] Marino J.P., Abe H. (1981). New stereospecific approach to steroid side chains: Conversion of dehydroepiandrosterone to cholesterol, isocholesterol, and their 15β-hydroxy derivatives. J. Am. Chem. Soc..

[44] Moon S., Stuhmiller L.M., Chadha R.K., McMorris T.C. (1990). Synthesis of dehydro-oogoniol and oogoniol: The adrenosterone route. Tetrahedron.

[45] Konno K., Fujishima T., Maki S., Liu Z., Miura D., Chokki M., Ishizuka S., Yamaguchi K., Kan Y., Kurihara M. (2000). Synthesis, biological evaluation, and conformational analysis of A-ring diastereomers of 2-methyl-1,25-dihydroxyvitamin D_3_ and their 20-epimers:  Unique activity profiles depending on the stereochemistry of the A-ring and at C-20. J. Med. Chem..

[46] Saito N., Honzawa S., Kittaka A. (2006). Recent results on A-ring modification of 1α,25-dihydroxyvitamin D_3_: Design and synthesis of VDR-agonists and antagonists with high biological activity. Curr. Top. Med. Chem..

[47] Hourai S., Fujishima T., Kittaka A., Suhara Y., Takayama H., Rochel N., Moras D. (2006). Probing a water channel near the A-ring of receptor-bound 1α,25-dihydroxyvitamin D3 with selected 2α-substituted analogues. J. Med. Chem..

[48] Honzawa S., Yamamoto Y., Hirasaka K., Takayama H., Kittaka A. (2003). Synthesis of A-ring synthon of 2α-substituted vitamin D_3_ analogues utilizing Grignard reaction towards methyl 2,3-anhydro-4,6-*O*-benzylidene-α-D-*manno*-pyranoside. Heterocycles.

[49] Saito N., Matsunaga T., Saito H., Anzai M., Takenouchi K., Miura D., Namekawa J., Ishizuka S., Kittaka A. (2006). Further synthetic and biological studies on vitamin D hormone antagonists based on C24-alkylation and C2α-functionalization of 25-dehydro-1α-hydroxyvitamin D_3_-26,23-lactones. J. Med. Chem..

[50] Kumagai G., Takano M., Shindo K., Sawada D., Saito N., Saito H., Kakuda S., Takagi K., Takimoto-Kamimura M., Takenouchi K. (2012). C15-Functionalized 16-ene-1α,25-dihydroxyvitamin D_3_ is a new vitamin D analog with unique biological properties. Anticancer Res..

[51] Baggiolini E.G., Iacobelli J.A., Hennessy B.M., Uskoković M.R. (1982). Stereoselective total synthesis of 1α,25-dihydroxycholecalciferol. J. Am. Chem. Soc..

